# ﻿ *Carex huancabambica* (Cyperaceae), a new species from the Peruvian and Ecuadorian Andes

**DOI:** 10.3897/phytokeys.265.161909

**Published:** 2025-10-31

**Authors:** Luis González-Gallego, Carmen Benítez-Benítez, Anton A. Reznicek, Asunción Cano, Nora H. Oleas, Santiago Martín-Bravo, Pedro Jiménez-Mejías

**Affiliations:** 1 Área de Botánica, Departamento de Biología Molecular e Ingeniería Bioquímica, Universidad Pablo de Olavide, Seville, Spain Universidad Pablo de Olavide Seville Spain; 2 Área de Botánica, Departamento de Biología Vegetal y Ecología, Facultad de Biología, Universidad de Sevilla, Seville, Spain Universidad de Sevilla Seville Spain; 3 University of Michigan Herbarium, Ann Arbor, MI, USA University of Michigan Herbarium Ann Arbor United States of America; 4 Laboratorio de Florística, Departamento de Dicotiledóneas, Museo de Historia Natural, Universidad Nacional Mayor de San Marcos, Lima, Peru Universidad Nacional Mayor de San Marcos Lima Peru; 5 Centro de Investigación de la Biodiversidad y Cambio Climático (BioCamb) e Ingeniería en Biodiversidad y Recursos Genéticos, Facultad de Ciencias de Medio Ambiente, Universidad Tecnológica Indoamérica, Quito, Ecuador Universidad Tecnológica Indoamérica Quito Ecuador

**Keywords:** Andes, Neotropics, new species, phylogenetics, *
Porocystis
*, South America, systematics, taxonomy

## Abstract

The Huancabamba Depression in Neotropical South America, a natural barrier between the Northern and Central Andes, serves as a refuge for high levels of angiosperm diversity. However, this biodiversity remains understudied, especially in complex and species-rich genera, such as *Carex* L. (Cyperaceae). This genus is notably underrepresented in taxonomic and systematic research on the Neotropics. In this study, we employed an integrative systematic approach combining molecular and morphological data to elucidate the taxonomic status of several *Carex* populations from Ecuador and northern Peru, which exhibit morphological affinities with the sect. Porocystis Dumort. (Castanea Clade). We conducted a phylogenetic analysis using two nuclear (ITS and ETS) and one plastid (*matK*) DNA regions and carried out a detailed morphological comparison with Neotropical relatives within the section. Both phylogenetic and morphological results supported the systematic distinctiveness of the focal populations. As a result, we describe a new species, *Carex huancabambica* Gonz.Gallego & Jim.Mejías, **sp. nov.** and provide its taxonomic treatment. This study contributes to disentangling the biodiversity of the genus *Carex* in the Neotropics.

## ﻿Introduction

The Neotropical Region harbours the richest plant diversity in the whole world, comprising approximately 90,000–110,000 species of seed plants, which represent around 37% of the world’s species (Antonelli and Sanmartín 2011). This region extends from central Mexico to northern Chile and central-western Argentina, covering the whole Central America, the majority of South America, the Antilles and South American Pacific Islands (Morrone 2014; Hermogenes De Mendonça and Ebach 2020). The remarkable levels of biodiversity of the Neotropics are reflected in the wide range of vegetation types, biomes and their underlying ecosystems. These include the tropical rainforests found in the Amazonian territories, the arid deserts expanding from northern Mexico, grass and tree savannahs, subtropical rainforests and subtropical dry fo­rests, areas of Mediterranean-like vegetation along the Pacific coast and the montane forests and high-altitude grasslands of the Andes Mountain range (Antonelli and Sanmartín 2011).

Within the South American Neotropics, the Andean cordilleras encompass the broadest range of biotic (e.g. interactions) and abiotic (e.g. temperature, rainfall, soil, topography) factors. This results in a great richness and diversity of flora distributed along an elevational gradient. The regionalisation of the tropical Andes comprises the Northern and Central Andes, extending approxi­mately 35 degrees of latitude from the north of the Sierra Nevada de Santa Marta (Colombia) and the Sierra Nevada de Mérida (Venezuela) to the northern­most regions of Salta and Jujuy in Argentina (Luteyn and Churchill 2000). The limit between the northern and the central parts of the cordilleras is given by the Huancabamba Depression (see below). The distinction between these two regions arises from differences in both abiotic and biotic factors, including, for example, the tectonic style – belonging to the northern and central volcanic zones, respectively – and the uplift history, inferred through paleoelevation estimates, based on modern climatic and geological indicators (Gregory-Wodzicki 2000). These differences are reflected in the current variation of the biodiversity found in both sides of the cordilleras and help delimit the northern and central regions of the Andes Mountains (Gregory-Wodzicki 2000; Weigend 2002).

The Huancabamba Depression, located in northern Peru and southern Ecuador, constitutes the lowest elevational zone (2,145 m at Abra de Porculla, Peru) in the entire Andean cordilleras and is recognised as a distinct phytogeographical region: the Amotape–Huancabamba Zone, that separates the Northern Andes from the Central Andes through the Río Chamaya and Río Marañón river systems (Berry 1982; Weigend 2002). This lower area constitutes a gap among high altitude Andean habitats and produces an important biogeographic turnover between the Northern and Central Andes. Conversely, this region serves as a convergence zone for taxa from both the Northern and Central Andes, acting as the southernmost range limit for northern taxa and the northernmost for southern taxa. The fragmented habitat and ecologically diverse ecosystems found in the Huancabamba Depression appear to have triggered speciation processes, as is reflected in the high plant species diversity and endemicity (Weigend 2002). For instance, species of *Nasa* Weigend (Loasaceae Juss.) occur in numbers six to eight times greater than in surrounding areas (Weigend 2002). These characteristics explain its consideration as a distinct biogeographical region and a major biodiversity hotspot, hosting approximately 318 endemic species of angiosperms (Weigend 2002).

*Carex* L. (Cyperaceae Juss.), with over 2,000 species, is the second most diverse genus of monocots after *Bulbophyllum* Thouars (Orchidaceae Juss.) and ranks among the five most species-rich genera of angiosperms (POWO 2025). It has a cosmopolitan distribution, with presence across all continents, except Antarctica, though it is largely absent from lowlands in tropical latitudes (POWO 2025). While *Carex* species predominantly inhabit wet environments in cold-temperate zones of the Northern Hemisphere (boreo-temperate regions), they are also found, to a lesser extent, in temperate areas of the Southern Hemisphere (Ball and Reznicek 2003; Martín-Bravo et al. 2019; Benítez-Benítez et al. 2021). In these southern regions, the genus has histori­cally been under­represented in taxonomic and systematic studies. Approximately 200 species of *Carex* have been described as native to South America, most of which are endemic to the continent (Morales-Alonso et al. 2024). In contrast to the extensive floristic treatments available for the genus in the Northern Hemisphere (e.g. *Carex* in the former USSR, Egorova (1999); Flora of North America, Ball and Reznicek (2003); Flora of China, Dai et al. (2010)), the South American species and clades have traditionally remained largely understudied, with only a few recent integrative taxonomic efforts addressing this knowledge gap (Jiménez-Mejías et al. 2020, 2021; Muñoz-Schüler et al. 2023; Morales-Alonso et al. 2024). Among the causes of this scenario are the limited accessi­bility of herbaria and digitisation of collections (which hinder taxo­nomic revisions or new descriptions), as well as the lack of regional taxonomic experts (Morales-Alonso et al. 2024).

Within the subgenus Carex, the predominantly American Castanea Clade comprises over 60 species, some of which are pending taxonomic re-evaluation. With only 40 species sampled in molecular phylogenetic studies to date (Roalson et al. 2021), the sectional delimitation of the clade remains unclear due to significant morphological heterogeneity. Traditional classification attempts (Hermann 1974; Ball and Reznicek 2003) recognised various sections within the Castanea Clade, the majority of which have been retrieved as polyphyletic in the latest phylogenetic framework classifications (Roalson et al. 2021). Nearly half of the species of the clade are comprised within sects *Longicaules* Mack. ex Reznicek and *Porocystis* Dumort. These two sections, along with the monospecific sect. Hirtifoliae Reznicek comprising North American *C. hirtifolia* Mack., represent the only monophyletic groups currently recognised within the clade (Roalson et al. 2021).

In North America, Mexico and Central America, Carex sect. Porocystis sensu stricto (sect. Porocystis hereafter) has been considered in Hermann’s (1974) revision of the genus in Mexico and Central America, the monographic treatment of the section in these regions by Reznicek and González-Elizondo (2001) and Flora of North America (Ball and Reznicek 2003). Species in this section are primarily native to North America, although some of them extend into the Neotropical regions of Mexico, Guatemala and South America and one species is also present in Eurasia (*C. pallescens* L.). They are morphologically commonly characterised by pubescent leaves and culms, as well as by mostly gynae­candrous terminal spikes and utricles often pubescent, but some variation in these characters is still observed among the species comprised within the section.

**Figure 1.**
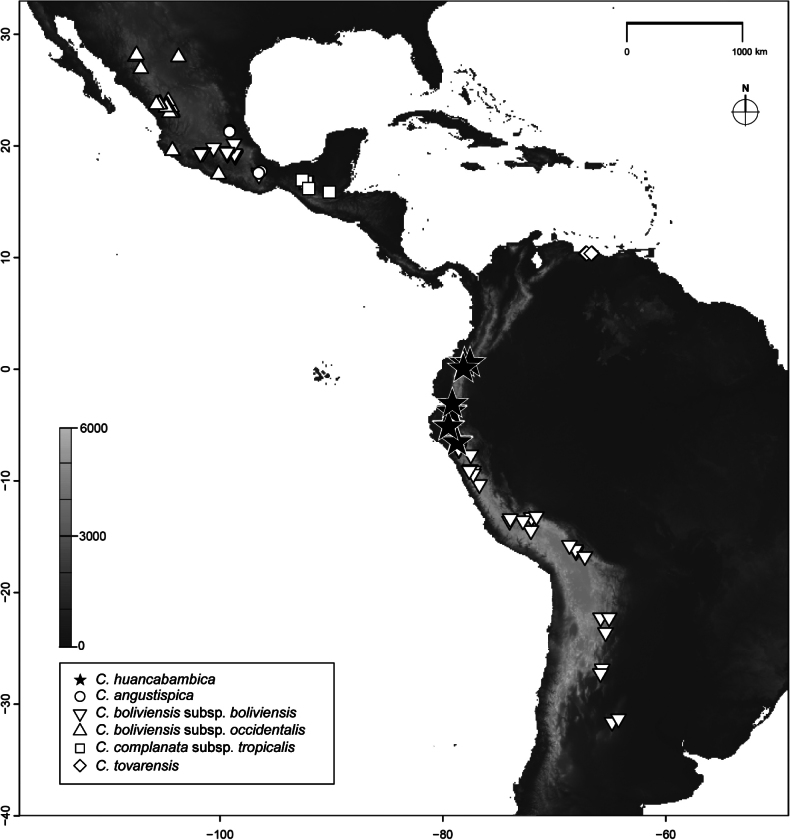
Distribution map representing the Neotropical species of Carex sect. Porocystis. Occurrence data (Suppl. material 2; Sanz-Arnal et al. 2025a, b) includes the studied specimens of *Carex huancabambica* sp. nov. (field collections and herbaria specimens examined, deposited at HUTI, QCA, QCNE, UPOS and USM).

To date, the Neotropical members of sect. Porocystis consist of four species and three subspecies (totalling seven taxa), clearly delimited both by clear-cut diagnostic morphological characters and by largely non-overlapping distribution ranges (Reznicek and Wheeler 1993; Reznicek and González-Elizondo 2001; Table 1; Fig. 1). Most taxa are distributed in North and Central America (Mexico and Guatemala) and the knowledge of the South American representatives of this section remains restricted to two species: the Venezuelan endemic *C. tovarensis*, and *C. boliviensis*, the type subsp. boliviensis remarkably disjunct between Mexico and the Andes (Table 1; Fig. 1). These taxa typically inhabit montane to alpine wet to moist environments, mostly in open habitats.

**Table 1.** Distribution range, habitat and altitude occupied by the Neotropical representatives of Carex sect. Porocystis. The newly described *C. huancabambica* is included for comparison. Data extracted from Reznicek and Wheeler (1993), and Reznicek and González-Elizondo (2001).

Species	*Carex angustispica* Reznicek & S.González	*Carex boliviensis* Van Heurck & Müll.Arg.		*Carex complanata* Torr. & Hook.	*Carex huancabambica* Gonz.Gallego & Jim.Mejías	*Carex tovarensis* Reznicek & G.A.Wheeler
		subsp. boliviensis	subsp. occidentalis Reznicek & S.González	subsp. tropicalis Reznicek & S.González		
**Distribution range**	Oaxaca (SW Mexico), with isolated occurrences in Querétaro (NE Mexico)	Disjunct in Mexico and the Andes, in Sierra Madre Oriental (C Mexico) and Transvolcanic Belt (S Mexico) and from N Peru to N Argentina	Sierra Madre Occidental, from S Chihuahua to Guerrero (NW to SW Mexico)	Central-American Cordillera, in Chiapas (SE Mexico) and Guatemala	N Andes, in N Peru and Ecuador, in the neighbouring regions around the Huancabamba Depression and north of it	Coastal range of northern Venezuela
**Habitat**	Subalpine open mesic habitats, including high-altitude grasslands, and open scrublands and pine forest	Subalpine to alpine moist and mesic open habitats, including grasslands, meadows and open scrublands	Montane to subalpine moist habitats in meadows and open pine and oak forest	Montane moist habitats in open scrublands and pine – oak forests	Montane to subalpine moist habitats, in meadows, grasslands and open forests	Montane open moist habitats, including high-altitude grasslands, and open scrublands
**Altitude (m)**	2,800–3,000	2,700–4,100	(2,000–) 2,500–3,200	1,600–2,800	2,900–3,900	2,000–2,300

In this study, we examined the taxonomic identity of several populations of a *Carex* taxon from northern South America that are morphologically assignable to sect. Porocystis. The specimens of study were collected from a number of localities in Ecuador (Provinces of Azuay, Carchi, Imbabura, Loja and Pichincha), as well as in northern Peru (Departments of Cajamarca and Piura) within the Amotape–Huancabamba Zone, immediately north of the northernmost limit of *C. boliviensis* in South America. Using morphological comparisons with closely-related taxa, combined with DNA-based phylogenetic analyses, we assessed whether these populations constitute a new species and determined their phylogenetic placement within the section. Accordingly, we proceed to describe it as *Carex huancabambica* Gonz.Gallego & Jim.Mejías.

## ﻿Materials and methods

### ﻿Molecular sampling

For the phylogenetic analyses conducted in this study, we included 27 acce­ssions belonging to selected taxa from the different sections included by Roalson et al. (2021) within the Castanea Clade (Suppl. material 1). As outgroup, we included *C. elliottii* from the Hirta Clade, a group closely related to the Castanea Clade (Roalson et al. 2021). These sequences were retrieved from previous *Carex* phylogenetic studies (Jiménez-Mejías et al. 2016; Martín-Bravo et al. 2019). To complete our sampling, we generated new sequence data from three populations of *C. huancabambica*, two specimens of *C. boliviensis* from Argentina and one from Peru and one specimen of C. complanata subsp. tropicalis from Guatemala (Suppl. material 1). The *C. huancabambica* material used for the molecular sampling were collected by the last two authors of this paper in two fieldwork campaigns in Peru and Ecuador. The vouchers were deposited at HUTI, UPOS and USM herbaria. Herbarium acronyms follow Thiers (2025).

### ﻿Molecular phylogenetic study

Phylogenetic relationships were reconstructed using three DNA barcoding regions commonly used in *Carex* phylogenetics at subgeneric and sectional levels: the nuclear ribosomal DNA (nrDNA) internal transcribed spacer (ITS), the nrDNA external transcribed spacer (ETS) and the chloroplast DNA (cpDNA) maturase K (*matK*) gene.

DNA extraction and sequence amplification followed Benítez-Benítez et al. (2021). All PCR products were sequenced externally by Macrogen (Madrid, Spain). Sequence chromatograms for each sample and marker were manually reviewed and processed using Geneious v.11.0.1 (Biomatters Ltd., Auckland, New Zealand; https://www.geneious.com). For each DNA region, an aligned matrix was constructed that included both the newly-obtained sequences and those retrieved from previous studies on *Carex* (Jiménez-Mejías et al. 2016; Martín-Bravo et al. 2019). First, the alignments were performed automatically using Muscle (Edgar 2004) and subsequently reviewed and edited manually. Informative insertions and deletions (indels) were manually coded in each matrix as additional binary characters. To determine the best-fit model of nucleotide substitution for each DNA region, we used jModelTest 2 2.1.10 v.20160303 (Darriba et al. 2012) following the Akaike Information Criterion (AIC; Nylander et al. (2004)). A singleton matrix was constructed selecting, for each taxon, the sequence with the least missing data per DNA region, resulting in a singleton dataset comprising 31 acce­ssions. However, for *C. boliviensis* and *C. huancabambica*, multiple sequences were sampled and included in the matrix to maximise the phylogenetic resolution in the involved branches. In total, 24 species were included in our phylogeny of the Castanea Clade, plus the outgroup sequence.

Phylogenetic reconstructions were performed using Bayesian Inference (BI) and Maximum Likelihood (ML) methods. Bayesian analyses were performed using MrBayes 3.2.7a (Ronquist et al. 2012), employing four simultaneous Markov Chain Monte Carlo chains run for five million generations, with trees sampled every 100 generations to estimate the posterior probability distribution. ML analyses were conducted using RAxML v.8.2.10 (Stamatakis 2014), with 100 bootstrap replicates. All analyses were run in CIPRES Science Gateway (Miller et al. 2015). Bayesian trees were visualised using FigTree v.1.4.4 (Rambaut 2008) and ML trees by using TreeGraph 2.15.0–887 beta (Stöver and Müller 2010). Clade support was considered strong at probability (PP) values greater than 0.8 and bootstrap support (BS) values greater than 60%.

### ﻿Morphological study

We examined representative material from the Neotropical members of Carex sect. Porocystis that were recovered in our phylogenies as closely related to *C. huancabambica* (see Results: Molecular and phylogenetic study). This included the collected specimens of *C. huancabambica* from Peru and Ecuador (Suppl. material 2), as well as herbarium specimens from the newly-described species and herbarium specimens of *C. boliviensis* (Suppl. material 2) and of *C. angustispica* (Suppl. material 2).

Material from the sampled populations of *C. huancabambica* (Suppl. material 2) was carefully examined for its description and compared with all morphologically close and phylogenetically related Neotropical species of sect. Porocystis (see Results: Molecular and phylogenetic study). The selection of diagnostic characters and morphological comparisons among species were based on both specialised literature on sect. Porocystis (Reznicek and González-Elizondo 2001) and herbarium specimens examined (Suppl. material 2). Measurements under 1 cm were taken using a binocular micrometer (Nikon SMZ645), except for macromorphological characters that were measured with a standard 30 cm ruler.

## ﻿Results

### ﻿Molecular and phylogenetic study

**Figure 2.**
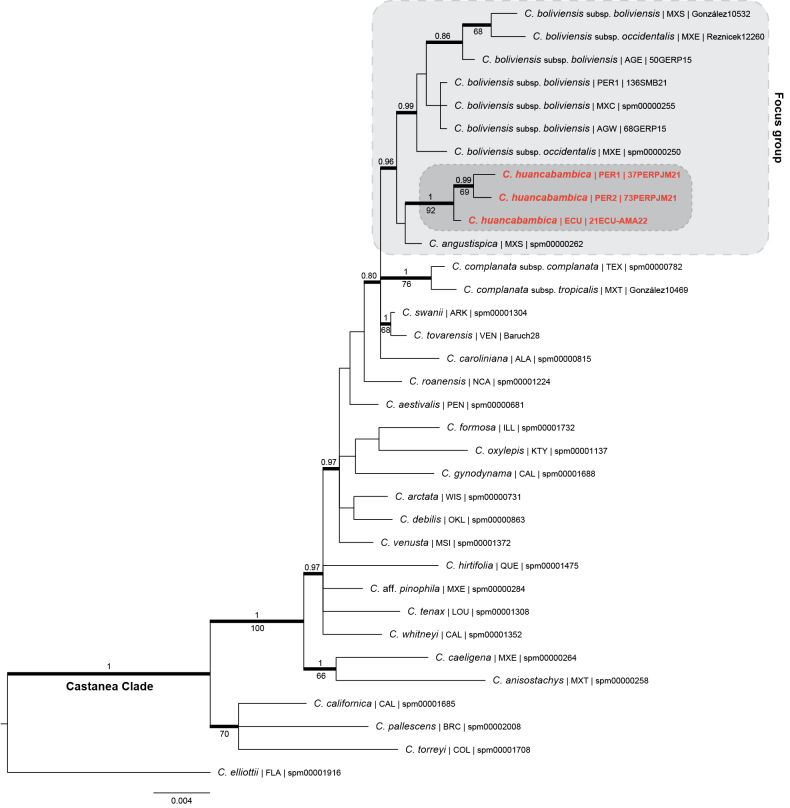
Bayesian phylogenetic tree obtained from the concatenation of nrDNA (ITS and ETS) and cpDNA (*matK*) sequences of species of the Castanea Clade including one tip per taxon and multiple tips per taxon for the focal populations, highlighted in grey. Both BI posterior probabilities (PP > 0.8) and ML bootstrap (BS > 60%) values are given above and below branches, respectively, for bold thick branches. The placement of *Carex huancabambica* sp. nov. is highlighted in red colour. Tip labels include the geographical origin of the specimen using TDWG level 3 region abbreviations (“botanical countries”; Brummitt (2001)) and the ID-specification of the specimen or voucher (Suppl. material 1).

The topology of the combined tree mostly agrees with that obtained from the nrDNA and cpDNA individual trees. For greater clarity, we describe the topology of the concatenated tree (Fig. 2). Our phylogenetic reconstruction shows a well-supported monophyletic group (PP = 1, BS < 60%), which is structured into two major subclades. The first subclade, which is moderately supported (PP < 0.8, BS = 70%), includes *C. californica*, *C. pallescens* and *C. torreyi*. The second subclade is strongly supported (PP = 1, BS = 100%) and corresponds to the rest of the included taxa of the Castanea Clade. *Carex caeligena* and *C. anisostachys* (PP = 1, BS = 66%) form a sister group to a larger, well-supported subclade (PP = 0.97, BS < 60%) that includes the focal Neotropical taxa of this study. In this subclade in particular, *C. huancabambica* yielded a supported monophyletic group with *C. boliviensis* and *C. angustispica*. The tree topology supported the recognition of *C. huancabambica* as a distinct species, recovered in a well-supported clade (PP = 1; BS = 92%). The only included sample of the Mexican *C. angustispica* was recovered as unresolved, whereas both Mexican and South American samples of the two *C. boliviensis* subspecies were recovered intermingled forming a monophyletic group (PP = 0.99, BS < 60%). The other Neotropical representatives of the group, C. complanata subsp. tropicalis and *C. tovarensis*, were excluded from that clade. Both samples of *Carex complanata* (subsp. boliviensis and subsp. boliviensis) formed a well-supported clade (PP = 1; BS = 76%). *Carex tovarensis*, on the other hand, was recovered in a well-supported clade with North American *C. swanii* (PP = 1; BS = 68%).

### ﻿Morphological study

**Figure 3.**
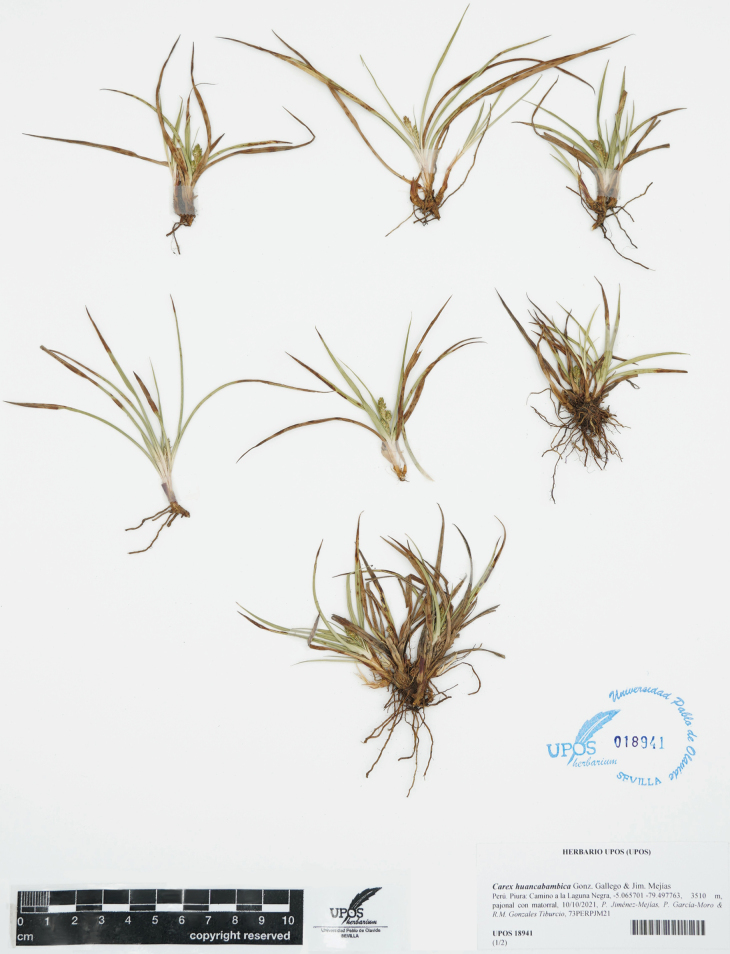
Holotype of *Carex huancabambica* sp. nov. (P. Jiménez-Mejías et al. 73PERPJM21) preserved at Universidad Pablo de Olavide Herbarium (UPOS 18941).

**Figure 4.**
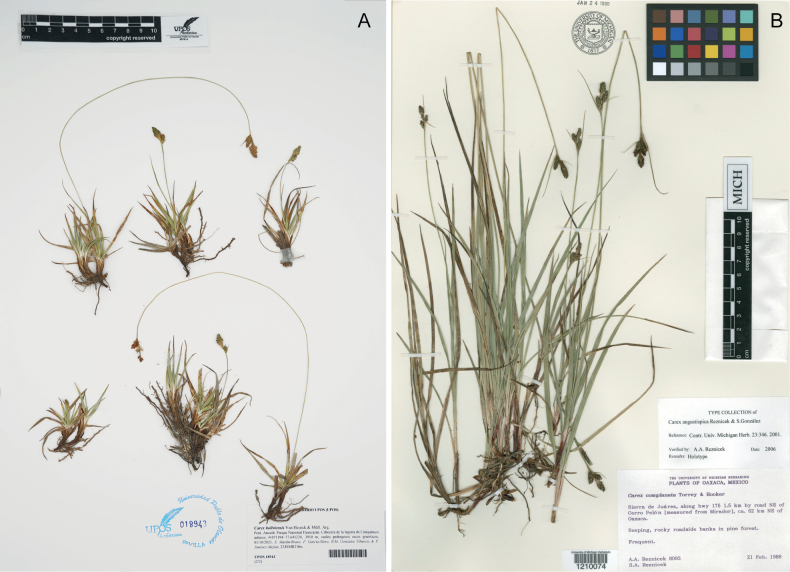
Representative material examined of: A. *Carex boliviensis* (S. Martín-Bravo et al. 224SMB21bis) housed at Universidad Pablo de Olavide Herbarium (UPOS 18943); note the elongated flexuous stems in the individuals with ripe utricles; B. Holotype of *Carex angustispica* (A. A. Reznicek 8093) housed at University of Michigan Herbarium (MICH 1210074).

**Figure 5.**
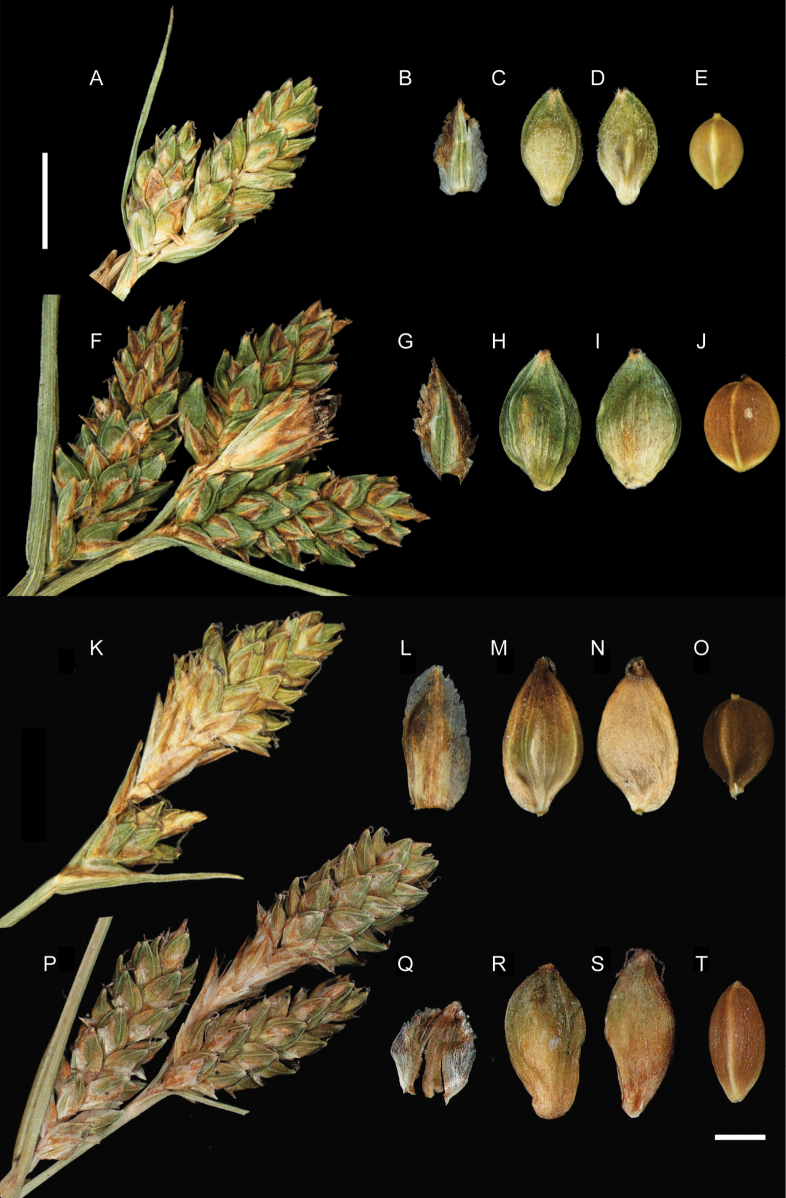
Comparative illustration of the main diagnostic morphological characters of *Carex huancabambica* (A–J), *Carex boliviensis* (K–O) and *Carex angustispica* (P–T). A–J. Details of inflorescence (A, F); glume (B, G); abaxial face of utricle (C, H); adaxial face of utricle (D, I); achene (E, J) of *Carex huancabambica* (A–E holotype, PERU, P. Jiménez-Mejías et al. 73PERPJM21, UPOS 18941; F–J P. Jiménez-Mejías et al. 37PERPJM21, UPOS 18942). K–O. Details of inflorescence (K); glume (L); abaxial face of utricle (M); adaxial face of utricle (N); achene (O) of *Carex boliviensis* (PERU, S. Martín-Bravo et al. 224SMB21bis, UPOS 18943). P–T. Details of inflorescence (P); glume (Q); abaxial face of utricle (R); adaxial face of utricle (S); achene (T) of *Carex angustispica* (holotype, MEXICO, A. A. Reznicek 8093, MICH 1210074). Scale bars: 4 mm (A, F, K, P); 1 mm (B–E, G–J, L–O, Q–T).

The detailed morphological examination and comparison of the diagnostic characters of *C. huancabambica* (Figs 3, 5A–J), *C. boliviensis* (Figs 4A, 5K–O) and *C. angustispica* (Figs 4B, 5P–T) revealed notable morphological affinities among the three taxa, but also clear differences in both qualitative and quantitative characters (Table 2).

**Table 2.** Comparison of the main diagnostic morphological characters among *C. huancabambica*, *C. boliviensis* and *C. angustispica*. Measurements for the first two were obtained following the procedures described in the Materials and Methods section, while data for *C. angustispica* were sourced from Reznicek and González-Elizondo (2001).

	*C. huancabambica*	*C. boliviensis*				*C. angustispica*
		subsp. boliviensis			subsp. occidentalis	
**Habit**	Erect, stiff culms	Decumbent or prostrate, wiry and flexuous culms			Robust, with erect culms, somewhat wiry and flexuous, arching or ± decumbent	Erect, stiff culms
**Fertile culm length (cm)**	< 0.50 (subacaulescent) to 25	3–64			35–87	(3–) 10–65
**Leaf length (cm)**	Up to 11	3.5–25				(2.5–) 5–28
**Leaf indumentum**	Glabrous to ± pubescent on abaxial surface, especially proximally and along the margins and veins	Pubescent basally or almost glabrous				Basally pilose
**Inflorescence length (mm)**	14–32	11–26 (–3.2)			(1–) 25–61	8–45
**Lateral spikes**	Pistillate, rarely staminate	Pistillate or gynaecandrous				Pistillate
**Terminal spike length (mm)**	10.0–15.5	(6–) 8–17			(9–) 15–30	ca. 14
**Spikes number**	(1–) 3–4	(1–) 2–4				2–4 (–5)
**Glume length (mm)**	1.7–2.5	1.8–2.3	1.8–3.4			1.6–2.4 (–2.6)
**Glume width (mm)**	1.0–1.5	1.1–2 (–2.2)				1.3–1.7
**Glume apex**	Obtuse, or acute to nearly acuminate	Obtuse to acute				Obtuse to acuminate
**Utricle length (mm)**	2.0–2.8	2.2–3.2 (–3.4)			3.0–4.1 (–4.6)	2.1–2.8
**Utricle width (mm)**	1.0–1.4	1.4–1.75				1.2–1.5
**Utricle shape**	Broadly elliptical to elliptical–obovate	Narrowly elliptical to ovate				Ovoid to obovoid
**Utricle indumentum**	Glabrous or loosely pilose on all its surface	Glabrous				Glabrous
**Achene length (mm)**	1.5–1.9	1.6–2.2		2.0–2.6		1.4–1.8
**Achene width (mm)**	0.9–1.3	1.1–1.5				1.1–1.3
**Achene shape**	Broadly elliptical to suborbicular	Broadly elliptical to suborbicular				Narrowly obovate to narrowly elliptical

## ﻿Discussion

Sources of phylogenetic and morphological evidence support the systema­tic distinctiveness of the studied Peruvian and Ecuadorian sect. Porocystis populations, validating the recognition of *C. huancabambica* as a new species (Figs 2, 3, 5A–J). The greatest morphological affinities of these popu­lations are observed with sect. Porocystis representatives *C. boliviensis* and *C. angustispica* according to the keys available in Reznicek and González-Elizondo (2001). However, clear-cut morphological differences between these taxa are also depicted (shape and indumentum of utricle and shape of achene; see Table 2). The distinctiveness is further supported by variation in geographic distribution and elevation (Table 1; Fig. 1). The mole­cular phylogeny also supported *C. huancabambica* as a different species from *C. boliviensis* and *C. angustispica* (Fig. 2).

Accordingly, we proceed to formally describe *C. huancabambica*. This discovery contributes to the taxonomic knowledge of the genus *Carex* in South America, enhancing our understanding of the understudied Neotropical representatives of the Castanea Clade and of sect. Porocystis, in particular.

All nomenclatural and taxonomic decisions in this study follow the rules and recommendations of the International Code of Nomenclature for algae, fungi and plants (ICN; Turland et al. (2018)).

### ﻿Taxonomic treatment

#### ﻿ Carex huancabambica

Taxon classificationPlantaePoalesCyperaceae

Gonz.Gallego & Jim.Mejías sp. nov.

6D5BCC02-1B33-5134-A1D5-94631E366961

urn:lsid:ipni.org:names:77369003-1

Figs 3, 5A–J

##### Diagnosis.

This species is superficially similar to *C. boliviensis*, from which it primarily differs by stiff short stems (wiry and flexuous in *C. boliviensis*), as well as by the utricle shape, broadly elliptical (ovate to narrowly elliptical in *C. boliviensis*). From the also closely-related *C. angustispica*, *C. huancabambica* differs in its smaller size, with culms from < 0.50 (subacaulescent) to 25 cm ((3–) 10–65 cm in *C. angustispica*) as well as in the shape of the utricles (ovoid to obovoid in *C. angustispica*).

##### Type.

Peru • Piura: Camino a la Laguna Negra, pajonal con matorral, 3,510 m alt., 05°03.9421'S, 79°29.8658'W, 10 Oct 2021, P. Jiménez-Mejías, P. García-Moro & R.M. Gonzales Tiburcio 73PERPJM21 (holotype: UPOS 18941!; isotype: USM!).

##### Specimens examined (paratypes).

Ecuador • Azuay: Totorococha–Mazan valley, Área Nacional de Recreación Cajas, paramo grassland, 3,600 m alt., 02°53.00000'S, 79°10.00000'W, 12 Sep 1987, P.M. Ramsay & P.J. Merrow-Smith 529 (QCA 206613!, QCNE 122100!); • Carchi: Provincia de Carchi en los cantones Tulcán, Espejo y Mira, Bosque Siempre Verde Montano Alto y Páramo de Frailejones en la zona de amortiguamiento de la Reserva Ecológica El Ángel, 3,625 m alt., 00°40.72676'S, 77°51.83465'W, 8 Oct 2011, V. Yunapanta & S. Chimbolema 167 (QCA 221878!); • same collection data as for preceding, V. Yunapanta & S. Chimbolema 169 (QCA 221873!); • Imbabura: Cotocachi Province [current Imbabura Province], slopes of Volcan Cotocachi, paramo grassland, 3,900 m alt., 00°35.00000'S, 78°20.00000'W, 11 Oct 1987, P.M. Ramsay & P.J. Merrow-Smith 815 (QCA 207075!); • Reserva Ecológica Cotacachi–Cayapas, faldas del Fuya–fuya, lagunas de Mojanda, crece en los pajonales que han sido dedicados a la ganadería, 3,819 m alt., 00°08.00000'S, 78°17.00000'W, 24 Oct 2000, L. Endara A. & M. Nonhebel 384 (QCA 36608!); • Loja: Cerca de Ramos Urku, carretera Loja–Cuenca, pastizal, 2,900 m alt., 03°40.5102'S, 79°16.0522'W, 28 Jul 2022, A. Morales-Alonso, P. Jiménez-Mejías, I. Masa-Iranzo, E. Sánchez 21ECU-AMA22 (UPOS 18944!, HUTI!); • Pichincha: Páramo de Mojanda. Between Laguna Grande and Laguna Negra, in dry pajonal, 3,700–3,800 m alt., 00°08.0000'S, 78°16.0000'W, 30 Jun 1985, S. Lægaard 54586 (QCA 36437!); Peru • Cajamarca: Carretera entre Chota y Cutervo, claros encharcados, 3,009 m alt., 06°27.10686'S, 78°45.48876'W, 7 Oct 2021, P. Jiménez-Mejías, P. García-Moro & R.M. Gonzales Tiburcio 36PERPJM21 (UPOS 18950!, USM!); • same collection data as for preceding, P. Jiménez-Mejías, P. García-Moro & R.M. Gonzales Tiburcio 37PERPJM21 (UPOS 18942!, USM!).

##### Morphological description.

Plants cespitose (Fig. 3). ***Fertile culms*** yellowish–­green, glabrous, < 0.50–25 cm long, but length variable within the same plant, erect, stiff, not flexuous, sometimes subacaulescent, with the culm concealed by the leaves, stems trigonous, sparsely antrorsely scabrid, especially at the distal part. ***Basal sheaths*** not fibrous, purplish-tinged. ***Leaves*** with sheaths membranous at top, hyaline, truncate to U-shaped, sparsely pilose, the open margins ciliate; ***ligule*** U-shaped, less than 0.5 mm to 1 mm, shorter than wide; ***blades*** up to 11 cm long, 2–4 mm wide, flat to M-shaped in cross-section, herbaceous, glabrous to more or less pubescent on abaxial surface, especially proximally and along the margins and veins, margins sparsely antrorsely scabrid, mainly distally. ***Inflorescences*** racemose, with (1–) 3–4 spikes (Fig. 5A, F), 14–32 mm long, 4–12 mm wide; proximal bracts leaf-like, 10–40 mm long, 9–21 mm wide, sparsely antrorsely scabrid, glabrous or ciliate on the abaxial face along margins and veins, sheathless or with a sheath up to 2–4 mm long, ciliate at the insertion with the stem; ***lateral spikes*** unisexual, pistillate (rarely staminate, seemingly androgynous in some undeveloped lateral spikes); peduncle sparsely antrorsely scabrid to glabrous; ***terminal spike*** gynae­candrous, 10.0–15.5 mm long, 3–4 mm wide, approx. 20–40 pistillate flowers per spike. ***Pistillate scales (glumes)*** 1.7–2.5 mm long, 1.0–1.5 mm wide, elliptical, glabrous, with apex obtuse or acute, sometimes nearly acuminate, rarely with shorter cilia at the apex, brownish distally and yellowing proximally, mid-vein lighter, margins hyaline (Fig. 5B, G). ***Utricles*** 2.0–2.8 mm long, 1.0–1.4 mm wide, broadly elliptical to elliptical–obovate, narrowly biconvex, uniformly brownish-green, glabrous (Fig. 5H, I) or loosely pilose on all its surface (Fig. 5C, D), with 2 prominent lateral nerves and sides nerveless or nearly so, tapering to the base and to the apex, beakless or with a very short inconspicuous truncate beak. Style withering; stigmas 3. ***Achenes*** trigonous, 1.5–1.9 mm long, 0.9–1.3 mm wide, broadly elliptical to suborbicular, almost filling entirely the utricles, tipped by a very short mucronate style remnant (Fig. 5E, J).

##### Distribution and habitat.

(Fig. 1) Ecuador (Provinces of Azuay, Carchi, Imbabura, Loja, Pichincha) and northern Peru (Departments of Cajamarca and Piura) in the context of the Amotape–Huancabamba Zone, separating the Northern from Central Andes. Present in open moist habitats on volcanic soils, at 2,900–3,900 m alt. Given the small size of the plant, additional populations of this species could exist and have been overlooked.

##### Phenology.

June–October.

##### Iconography.

Figs 3, 5A–J.

##### Conservation status.

This species is currently known from seven populations, covering a distribution range within an extent of occurrence (EOO) of 60,219 km^2^ and an area of occupancy (AOO) of 36 km^2^ (based on IUCN default cell width of 2 km^2^ and estimated according to the proximity of the closest populations studied). This geographic range suggests the application of criterion B2 for the Endangered category (EN; threshold of < 500 km^2^ for AOO; IUCN (2024)). While the number of locations (< 10) would indicate the potential application of the Vulnerable (VU) category under criterion B2, the lack of data on the demographic tendency of the studied populations and the fact that probable additional overlooked populations of this species exists, prevent the application of any threatened categories, as no more than one sub-condition can be fulfilled (at least two are needed). Based on the available data from the studied material (Suppl. material 2) and considering the restricted AOO, we hypothesise that *C. huancabambica* should be currently classified as Data Deficient (DD) according to the global IUCN conservation categories. Therefore, at present, there is not sufficient information to conduct a complete assessment of the conservation status of this species.

##### Etymology.

The species epithet, *huancabambica*, is derived from the Huancabamba Depression within the Amotape–Huancabamba Zone in the Andes, that extends between Piura and Cajamarca in northern Peru to Loja in southern Ecuador.

##### Systematic and biogeographic notes.

The description of this new species highlights the taxonomic complexity within Carex sect. Porocystis, especially among its understudied Neotropical taxa. The phylogenetic analyses (Fig. 2) reveal that *C. angustispica* is among the closest related taxa to *C. huancabambica*, despite its exclusive occurrence in the Mexican States of Oaxaca and Querétaro and its absence from South America (Reznicek and González-Elizondo 2001). Additional phylogenetic resolution is still needed to understand the placement of *C. angustispica* within sect. Porocystis. *Carex boliviensis* appears to be the sister taxon to the *C. angustispica*-*C. huancabambica* clade and inhabits similar mountain environments as *C. huancabambica*, but south of the Amotape–Huancabamba Zone, through the Central and the north of the Southern Andes, reaching higher altitudes (up to 4,100 m). No shared localities have been recorded between C. boliviensis subsp. boliviensis and *C. huancabambica*, the latter only being recorded immediately north of the distribution limit of C. boliviensis subsp. boliviensis in South America. Only a single locality in Oaxaca, southwest Mexico, is known to harbour more than one species of sect. Porocystis (*C. angustispica* and C. boliviensis subsp. boliviensis; Reznicek and González-Elizondo (2001)).

### ﻿Identification key to the Neotropical taxa of Carex sect. Porocystis

The following key has been modified from that in Reznicek and González-Elizondo (2001) to accommodate the distinction of *C. huancabambica* from the other Neotropical species of sect. Porocystis.

**Table:** 

1a	Fertile culms more or less flexuous; utricles narrowly elliptical to ovate; lateral spikes pistillate or gynaecandrous, rarely staminate	**2a**
2a	Inflorescences 1.1–2.6 (–3.2) cm long with terminal spikes (6–) 8–17 mm long, the staminate portion usually less than half the length of the spike; the lowest bract often shorter than or about equalling the inflorescence, rarely longer	**Carex boliviensis subsp. boliviensis**
2b	Inflorescences (1–) 2.5–6.1 cm long with terminal spikes (9–) 15–30 mm long, the staminate portion often half the length of the spike or longer; the lowest bract often longer than the inflorescence	**Carex boliviensis subsp. occidentalis**
1b	Fertile culms stiff, erect, straight or arched; utricles elliptical to obovate; lateral spikes pistillate, rarely staminate	**3a**
3a	Utricle elliptical; achene broadly elliptical to suborbicular or obovate	**4a**
4a	Utricles trigonous rhombic–elliptic, sparsely pilose at least in the distal two-thirds, conspicuously red-dotted; achenes elliptic to obovate	***Carex tovarensis***
4b	Utricles narrowly biconvex, broadly elliptical to elliptical–obovate, uniformly brownish-green, glabrous or loosely pilose, without red dots; achenes broadly elliptical to suborbicular	***Carex huancabambica***
3b	Utricle obovate; achene narrowly elliptical to narrowly ovate or obovate	**5a**
5a	Length of fertile culms (3–) 10–65 cm long; pistillate scales 1.2–1.6 times as long as wide, obtuse to acute, sometimes minutely cuspidate; achenes 1.4–1.8 mm long; pistillate portion of terminal spikes 2.8–5.1 mm wide	***Carex angustispica***
5b	Length of fertile culms (20–) 35–86 cm long; pistillate scales 1.6–3.3 times as long as wide, obtuse to acuminate-awned; achenes 1.7–2.2 mm long; pistillate portion of terminal spikes (4.4–) 4.8–7.3 mm wide	**Carex complanata subsp. tropicalis**

## ﻿Conclusions

Integrative approaches, based on multiple lines of evidence, are essential for resolving complex systematic scenarios. The biogeographical position, along with the geological and climatic characteristics of the Huancabamba Depression in the Andes of southern Ecuador and northern Peru, have led to high levels of diversification in the region, particularly in angiosperm diversity, and serves as a limit between the northern and central parts of the Andes. In this study, describe a new species, *Carex huancabambica* sp. nov. and contribute to disentangling the taxonomy and systematics of the genus *Carex* in South America, with a particular emphasis on the understudied Neotropical representatives of sect. Porocystis s.s. within the Castanea Clade.

## Supplementary Material

XML Treatment for Carex huancabambica

## ﻿Additional information

### Use of AI

No use of AI was reported.

### Author ORCIDs

Luis González-Gallego https://orcid.org/0009-0006-1643-7292

Carmen Benítez-Benítez https://orcid.org/0000-0003-4956-0343

Anton A. Reznicek https://orcid.org/0000-0002-9467-6225

Asunción Cano https://orcid.org/0000-0002-5759-4650

Nora H. Oleas https://orcid.org/0000-0002-1948-4119

Santiago Martín-Bravo https://orcid.org/0000-0003-0626-0770

Pedro Jiménez-Mejías https://orcid.org/0000-0003-2815-4477

### Data availability

All of the data that support the findings of this study are available in the main text or Supplementary Information.

## Supplementary materials

Supplementary material 1 List of sequenced specimens used for the phylogeny Data type xlsx Explanation note List of sequenced specimens used for the phylogeny (Fig. 2) with their accession numbers included, including the newly-sequenced material of the taxa of study, highlighted in bold and the previously published sequences (Jiménez-Mejías et al. 2016; Martín-Bravo et al. 2019). https://binary.pensoft.net/file/1455119This dataset is made available under the Open Database License (http://opendatacommons.org/licenses/odbl/1.0/). The Open Database License (ODbL) is a license agreement intended to allow users to freely share, modify, and use this Dataset while maintaining this same freedom for others, provided that the original source and author(s) are credited.Luis González-Gallego, Carmen Benítez-Benítez, Anton A. Reznicek, Asunción Cano, Nora H. Oleas, Santiago Martín-Bravo, Pedro Jiménez-Mejías

Supplementary material 2 List of studied material used for the morphological part of the present study Data type xlsx Explanation note List of studied material used for the morphological part of the present study (Figs 3–5), including all the specimens with coordinate data (Sanz-Arnal et al. 2025a, b) used to build the distribution map of the Neotropical species of sect. Porocystis (Fig. 1). Information of taxon, authors, country (TDWG level 3 region abbreviations (“botanical countries”; Brummitt (2001)), locality of collection, herbarium and voucher number, collector and code of collector is included. Examined specimens are marked with !. https://binary.pensoft.net/file/1455120This dataset is made available under the Open Database License (http://opendatacommons.org/licenses/odbl/1.0/). The Open Database License (ODbL) is a license agreement intended to allow users to freely share, modify, and use this Dataset while maintaining this same freedom for others, provided that the original source and author(s) are credited.Luis González-Gallego, Carmen Benítez-Benítez, Anton A. Reznicek, Asunción Cano, Nora H. Oleas, Santiago Martín-Bravo, Pedro Jiménez-Mejías
